# Community-Acquired Legionnaires’ Disease Presenting With Gastrointestinal and Neurological Symptoms Without Respiratory Complaints in an Elderly Male

**DOI:** 10.7759/cureus.95814

**Published:** 2025-10-31

**Authors:** Sourav K Saha, Nedaa Haddad

**Affiliations:** 1 General Internal Medicine, Aneurin Bevan University Health Board, Ystrad Mynach, GBR; 2 Geriatric Medicine, Aneurin Bevan University Health Board, Ystrad Mynach, GBR

**Keywords:** atypical pneumonia, community-acquired infection, delirium, gastrointestinal symptoms, legionella pneumophila, legionnaires’ disease

## Abstract

Legionnaires’ disease, caused by Legionella pneumophila (L. pneumophila), is a recognized but often underappreciated cause of community-acquired pneumonia, especially in elderly patients with multiple comorbidities who may present with atypical and predominantly systemic features. We report the case of a 78-year-old man with a history of chronic obstructive pulmonary disease, chronic kidney disease, non-alcoholic fatty liver disease, and hypertension, who presented to the medical assessment unit with fever, acute confusion, diarrhea, vomiting, and headache but notably without any respiratory complaints. On admission, he was febrile, disoriented, and hemodynamically stable, with the confusion, urea, respiratory rate, blood pressure, age ≥65 (CURB-65) pneumonia severity score of three indicating high risk of mortality, and a 4 A’s Test (4AT) score of six confirming delirium. Laboratory investigations revealed leukocytosis, markedly elevated C-reactive protein, hyponatremia, and renal impairment, while blood cultures were negative after 48 hours of incubation. Chest radiography demonstrated bilateral infiltrates, despite the absence of cough or dyspnea. Urinary antigen and sputum polymerase chain reaction (PCR) confirmed L. pneumophila serogroup 1 infection. The patient was initially treated empirically with broad-spectrum antibiotics but was narrowed to oral levofloxacin for 14 days following confirmation of the diagnosis, with full resolution of neurological and systemic symptoms. A public health investigation identified a contaminated domestic hot tub as the likely source of infection. This case underscores the importance of maintaining a high index of suspicion for Legionnaires’ disease in elderly patients presenting with delirium and gastrointestinal symptoms, the critical role of rapid diagnostic testing in guiding appropriate therapy, and the need for environmental surveillance and intervention to prevent further sporadic community-acquired cases.

## Introduction

Legionnaires’ disease, caused by Legionella pneumophila (L. pneumophila), is an important but often underdiagnosed cause of community-acquired pneumonia, particularly in elderly individuals with multimorbidity [1,2]. Although classically associated with respiratory symptoms such as cough, dyspnea, and chest discomfort, Legionella infections can present atypically, especially in older or immunocompromised patients [3-5]. Gastrointestinal symptoms, including diarrhea, nausea, and vomiting, as well as neurological manifestations such as confusion, headache, and delirium, may dominate the clinical picture [3,4], potentially delaying recognition and treatment [1,2].

Transmission of Legionella occurs via inhalation of contaminated aerosols from water sources, including cooling towers, showers, decorative fountains, and increasingly, domestic hot tubs [6-9]. Despite well-publicized travel-associated outbreaks, sporadic community-acquired infections continue to occur, often linked to poorly maintained or contaminated water systems [7-11]. Legionnaires’ disease is a notifiable condition in many countries, including the UK, due to its public health implications and potential for outbreaks [10,11].

Early diagnosis relies on a high index of suspicion and the use of rapid diagnostic tools such as urinary antigen testing and polymerase chain reaction (PCR) [2,6,12], which facilitate timely initiation of appropriate therapy. Fluoroquinolones or macrolides are recommended as first-line treatments due to their excellent intracellular activity [2,13].

This case illustrates the diagnostic challenges of community-acquired L. pneumophila infection presenting with predominantly gastrointestinal and neurological symptoms and no initial respiratory complaints. While delirium and hyponatremia-related gastrointestinal features are recognized manifestations, the absence of respiratory symptoms is uncommon and may delay diagnosis. Clinicians should maintain a high index of suspicion for Legionella in elderly patients with systemic symptoms, even when pulmonary involvement is only evident on imaging [1-3,7-9], as age-related immunosenescence can blunt typical respiratory signs such as cough.

## Case presentation

A 78-year-old man, a former smoker with a history of chronic obstructive pulmonary disease (COPD), chronic kidney disease, non-alcoholic fatty liver disease, and hypertension, presented to the emergency department with a three-day history of fever, acute confusion, diarrhea, vomiting, and headache [3,4]. Notably, he denied cough, chest pain, or shortness of breath.

Initial assessment

On initial examination, the patient was febrile (38.6 °C), disoriented, and demonstrated acute cognitive impairment, with a 4 A’s Test (4AT) score of six, confirming delirium [2]. He was hemodynamically stable, with normal blood pressure and heart rate, and no signs of respiratory distress were observed at presentation. The patient reported no recent travel, sick contacts, or exposure to high-risk environments. Neurological and gastrointestinal symptoms predominated, including headache, nausea, and diarrhea, which drew early attention to systemic presentation rather than classical respiratory infection.

Interpretations of chest radiographs and lab results

**Figure 1:**
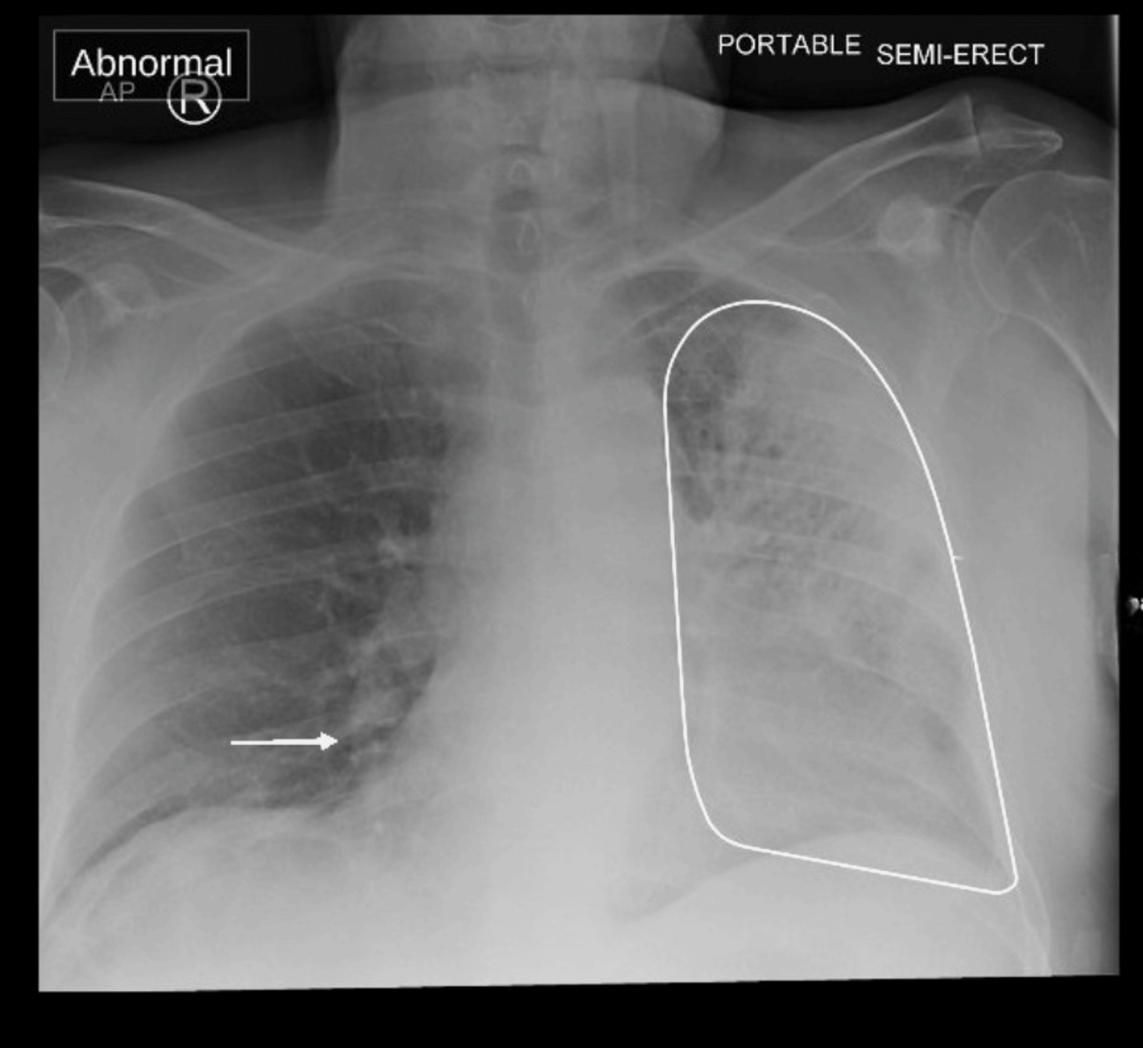
Chest radiograph on admission

Chest radiography revealed consolidation in the left upper lobe, with additional patchy opacities in the right lower lobe, suggestive of multilobar involvement (Figure 1), which is common in Legionella pneumonia and often correlates with more severe disease [2,5].

Laboratory investigations demonstrated leukocytosis (13.4 × 10⁹/L) (Table 1) and a markedly elevated C-reactive protein (CRP, 349 mg/L) (Table 2), reflecting acute infection and systemic inflammation consistent with severe community-acquired pneumonia [1,2]. Elevated CRP levels reflect a heightened systemic inflammatory response mediated by cytokines such as interleukin-6 (IL-6) and tumor necrosis factor alpha (TNF-α), which drive hepatic CRP production and leukocyte activation. This exaggerated inflammatory response correlates with disease severity, tissue injury, and risk of organ dysfunction, particularly when associated with multilobar involvement, bacteremia, and an increased need for intensive care admission. Thus, the markedly raised CRP and leukocytosis in this patient not only signify acute infection but also serve as surrogate indicators of severe pneumonia, consistent with the patient’s clinical and radiological findings. Serum sodium was reduced (132 mmol/L) (Table 2), and renal function tests showed creatinine 134 µmol/L and urea 10.8 mmol/L (Table 2), indicating acute kidney injury (AKI), which can occur secondary to severe infection, dehydration, or direct renal involvement from Legionella [2,5]. Additionally, his confusion, urea, respiratory rate, blood pressure, age ≥65 (CURB-65) pneumonia severity score was 3, indicating a high risk of mortality.

**Table 1:** Laboratory results on admission

Test	Description	Result	Unit	Flag	Reference Range
WBC	White blood cell (WBC) count	13.4	x10^9/L	H	4.0 - 11.0
HB	Haemoglobin (Hb)	125	g/L	L	130 - 180
PLTS	Platelet (PLT) count	122	x10^9/L	L	150 - 400
RBC	Red blood cell (RBC) count	3.63	x10^12/L	L	4.50 - 6.00
HCT	Haematocrit (Hct)	0.37	L/L	L	0.40 - 0.52
MCV	Mean cell volume (MCV)	102	fL	H	80 - 100
MCH	Mean cell haemoglobin (MCH)	34.4	pg	H	27.0 - 33.0
RDW-CV	Red cell distribution width (RDW)	12.9	%		11.0 - 14.8
NEUT	Neutrophil count	12.5	x10^9/L	H	1.7 - 7.5
LYMPH	Lymphocyte count	0.5	x10^9/L	L	1.0 - 4.5
MONO	Monocyte count	0.4	x10^9/L		0.2 - 0.8
EOS	Eosinophil count	0.0	x10^9/L		0.0 - 0.4
BASO	Basophil count	0.0	x10^9/L		0.0 - 0.1
NRBC	Nucleated red blood cell (NRBC) count	0.0	x10^9/L		

**Table 2:** Laboratory results on admission

Test	Description	Result	Unit	Flag	Reference Range
CA	Calcium	2.22	mmol/L		2.20 - 2.60
CALC	Calcium (adjusted)	2.35	mmol/L		2.20 - 2.60
TP	Protein	67	g/L		60 - 80
ALB	Albumin	29	g/L	L	35 - 50
GLOB	Globulin	38	g/L		22 - 43
PHOS	Phosphate	0.67	mmol/L	L	0.80 - 1.50
ALP	Alkaline phosphatase	67	U/L		30 - 130
CRP	C-reactive protein (CRP)	349	mg/L	H	<10
BILI	Bilirubin	17	umol/L		<21
ALT	Alanine transaminase	27	U/L		<41
Urea	Urea	10.8	mmol/L	H	2.5 - 7.8
NA	Sodium	132	mmol/L	L	133 - 146
K	Potassium	3.2	mmol/L	L	3.5 - 5.3
UOP	Urea	10.8	mmol/L	H	2.5 - 7.8
CREAT	Creatinine	134	umol/L	H	58 - 110
EGFR	Estimated Glomerular Filtration Rate	45	ml/min/1.73m2		

Blood culture was negative after 48 hours, as expected given the fastidious growth requirements of Legionella species [6]. A urinary Legionella antigen test was positive on two separate occasions, and PCR testing of sputum confirmed the presence of L. pneumophila serogroup 1 [1,5,6]. Viral testing, including influenza A, influenza B, and COVID-19, was negative (Table 3). A urinary Streptococcus pneumoniae (S. pneumoniae) antigen test was not performed; however, blood cultures and routine microbiological investigations did not suggest S. pneumoniae infection. Although viral-bacterial co-infections can occasionally present with diarrhea, hyponatremia, and pneumonia in the absence of cough, no evidence of such co-infection was observed in this patient. We acknowledge the absence of a urinary S. pneumoniae antigen test as a limitation, though the clinical and laboratory findings strongly support Legionella as the causative pathogen.

**Table 3:** Results of viral testing

Viral Test	Result
SARS-CoV-2	Not Detected
Influenza A	Not Detected
Influenza B	Not Detected

Based on the 2019 American Thoracic Society/Infectious Diseases Society of America (ATS/IDSA) guidelines, the patient met the criteria for severe community-acquired pneumonia, fulfilling three minor criteria: multilobar infiltrates on imaging, confusion, and elevated serum urea (10.8 mmol/L; equivalent to BUN 30.2 mg/dL) [2].

It is to be noted that, during the inpatient stay, his other bloods, including WCC, CRP, and renal function, also showed significant improvement after initiation of appropriate treatment (Table 4).

**Table 4:** Improved laboratory results while inpatient stay

Test	Result	Unit	Reference Range	Status
White Blood cell (WBC) Count	7.6*10^9^	L	4.0 – 11.0	Normal
C-reactive protein (CRP)	143	mg/L	<10	High (H)
Sodium (NA)	138	mmol/L	133 - 146	Normal
Potassium (K)	3.5	mmol/L	3.5 - 5.3	Normal
Urea (UOP)	9.5	mmol/L	2.5 - 7.8	High (H)
Creatinine (CREAT)	86	umol/L	58 - 110	Normal
Estimated Glomerular Filtration Rate (eGFR)	75	ml/min/1.73m^2^	(No range provided)	(No range provided)

**Figure 2:**
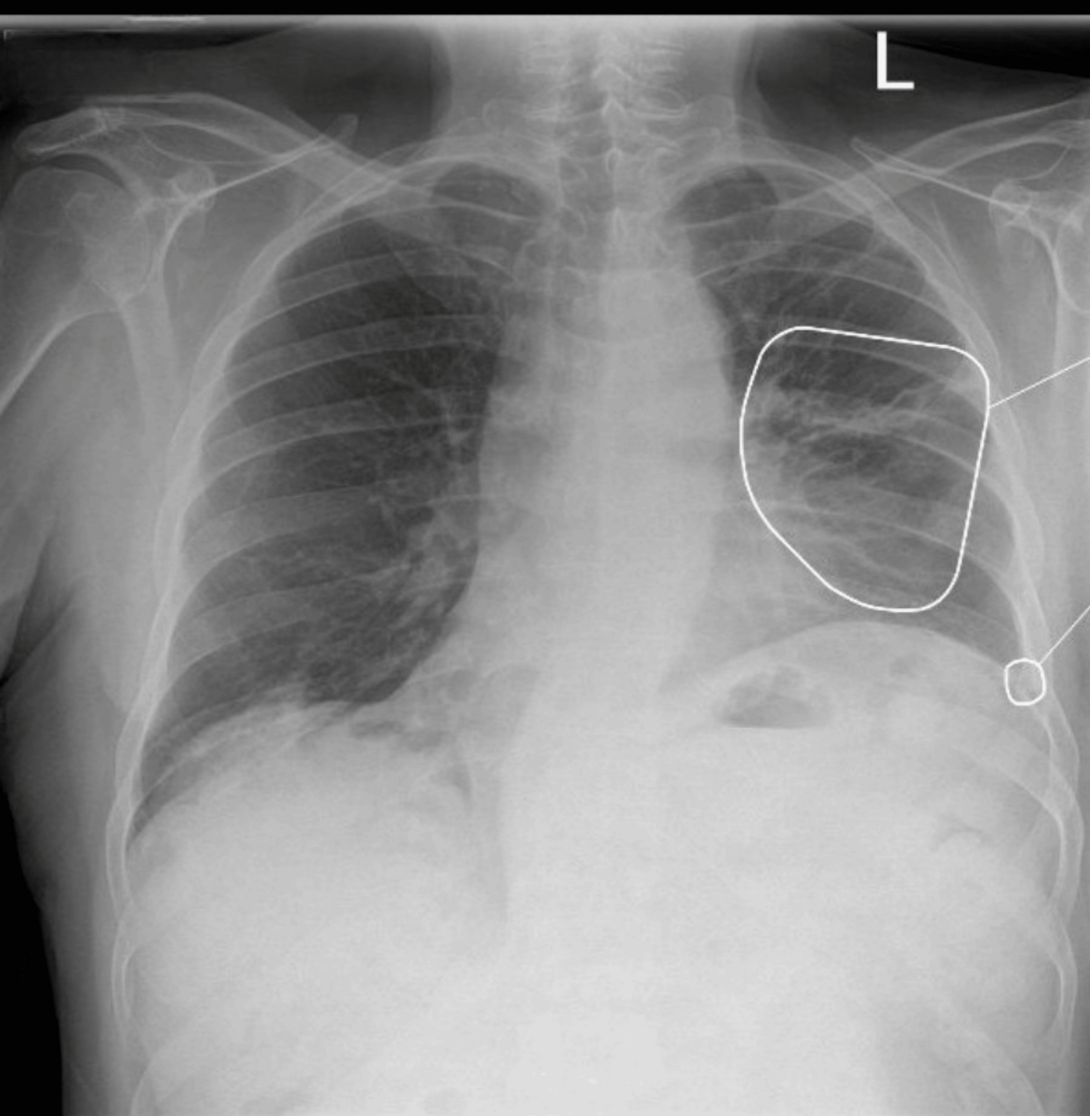
Follow up chest radiograph

Follow-up imaging approximately four weeks later demonstrated significant radiological improvement; right lower lobe infiltration resolved, along left-sided infiltration showed a significant size reduction (Figure 2). 

Management

Initial empiric therapy with intravenous piperacillin-tazobactam was initiated for presumed sepsis of unknown source. This was subsequently switched to amoxicillin and clarithromycin following radiographic findings, and the patient was treated as having community-acquired pneumonia [2]. Upon confirmation of Legionella infection, therapy was narrowed to oral levofloxacin for 14 days [2,13]. The longer antibiotic course was selected specifically due to L. pneumophila infection.

While the 2019 IDSA/ATS guidelines recommend a five to seven-day course for most community-acquired pneumonias [2], Legionella infections require extended therapy because of the organism’s intracellular persistence, slower clinical response, and higher relapse risk with shorter regimens. Fluoroquinolones achieve excellent intracellular penetration, and a 10-14-day course is recommended for immunocompetent adults, extending up to 21 days in immunocompromised or severe cases. Accordingly, our patient’s 14-day course of oral levofloxacin aligns with guideline-based management for severe Legionnaires’ disease.

Clinical course and outcome

The patient’s neurological and gastrointestinal symptoms began to improve within 48 hours of initiating targeted levofloxacin therapy. His delirium resolved with targeted antimicrobial therapy and supportive care, including hydration, monitoring of electrolytes, and minimizing environmental stimuli. No specific pharmacologic intervention for delirium was required, as by day three, his cognition had returned to baseline, gastrointestinal symptoms had completely resolved, and renal function nearly normalized. He remained clinically stable throughout his inpatient stay and was discharged after 06 days, with follow-up chest imaging planned at six weeks.

Public health investigation

A public health investigation identified the patient’s poorly maintained domestic hot tub as the most probable source of infection, based on recent exposure within the incubation period and absence of alternative environmental exposures. Although environmental sampling was not undertaken, the hot tub’s inadequate maintenance and compatible clinical presentation supported its implication as the likely source of community-acquired Legionella infection [7-9], highlighting the importance of community water reservoirs in sporadic Legionnaires’ disease.

## Discussion

This case underscores the complexity and diagnostic challenges of community-acquired Legionella pneumonia, particularly when patients present with atypical systemic manifestations rather than classical respiratory symptoms [1,3-5]. L. pneumophila is a significant but under-recognized pathogen in community-acquired pneumonia (CAP), with a predilection for older adults, immunocompromised individuals, and those with chronic comorbidities such as cardiovascular disease, diabetes mellitus, or chronic kidney disease [1,2]. Early recognition is challenging due to the heterogeneous presentation and non-specific laboratory abnormalities.

Our patient initially presented with confusion, diarrhea, vomiting, and headache, but notably lacked respiratory complaints [3,4]. Such non-respiratory features are increasingly documented in the literature, particularly among elderly patients, where neurological manifestations such as delirium and gastrointestinal involvement are prominent [3-5,14]. Reports have also described broader atypical presentations, including neurological, cardiac, and gastrointestinal symptoms, underscoring the diverse clinical spectrum of Legionella infection [14].

Imaging studies are pivotal in such presentations to identify subclinical pulmonary involvement. In this case, chest radiography revealed left upper lobe consolidation alongside patchy right lower lobe opacities, consistent with an atypical pneumonia pattern [2]. Although these findings are not pathognomonic for Legionella infection, they support the diagnosis in the context of systemic symptoms and relevant exposure risk factors. Historically, the term “atypical pneumonia” referred to infections caused by pathogens such as L. pneumophila, Mycoplasma pneumoniae, and Chlamydia pneumoniae, which are difficult to detect using standard culture techniques. However, according to the IDSA/ATS guidelines, the distinction between “typical” and “atypical” pneumonia is primarily clinical-based on extrapulmonary features and a subacute course-rather than radiographic characteristics [2].

Laboratory investigations revealed leukocytosis, markedly elevated CRP, hyponatremia, and impaired renal function, which are consistent with Legionella infection but remain non-specific [1,2,12]. Serum sodium was reduced (132 mmol/L), consistent with hyponatremia, a biochemical abnormality commonly associated with Legionella infection-attributable to the syndrome of inappropriate antidiuretic hormone secretion (SIADH) triggered by L. pneumophila, leading to water retention and dilutional hyponatremia [1,2]. Renal impairment may reflect dehydration due to gastrointestinal losses or direct renal involvement mediated by the systemic inflammatory response [1,2].

According to the 2019 IDSA/ATS guidelines, urinary antigen testing for L. pneumophila and S. pneumoniae is recommended for patients with severe CAP or those with epidemiologic risk factors for Legionella exposure [2]. In this case, the patient met three minor severity criteria-multilobar infiltrates, confusion, and elevated serum urea (10.8 mmol/L, equivalent to BUN 30.2 mg/dL)-thereby fulfilling the definition of severe pneumonia. Consequently, Legionella testing was clinically warranted and consistent with ATS/IDSA recommendations [2].

Rapid and accurate diagnostic testing was critical in this case. Urinary antigen testing, followed by confirmatory polymerase chain reaction (PCR), established the presence of L. pneumophila serogroup 1 [6]. Early identification enabled targeted antimicrobial therapy, which is essential because standard empiric β-lactam therapy is ineffective against intracellular Legionella species. Oral levofloxacin, a fluoroquinolone with excellent intracellular penetration and bactericidal activity, was administered in accordance with guideline recommendations and was associated with rapid clinical improvement [2,13]. The efficacy of fluoroquinolones in Legionella infection is attributed to their ability to achieve high intracellular concentrations, effectively eradicating bacteria residing within alveolar macrophages.

Beyond individual patient management, this case has significant public health implications. Although outbreaks of Legionnaires’ disease tend to attract attention, sporadic cases originating from domestic or community sources remain clinically and epidemiologically important [7-9]. Identification of a contaminated domestic hot tub as the infection source highlights the necessity of routine environmental maintenance, risk assessment, and public health reporting [10,11,15]. Stagnant water, warm temperatures, and inadequate disinfection promote Legionella proliferation, increasing the risk of aerosolized transmission [12,15]. As Legionella infection is a notifiable disease, timely recognition and communication with public health authorities are essential to prevent secondary cases and mitigate community spread.

In conclusion, this case illustrates the multifaceted challenges of diagnosing and managing Legionnaires’ disease in elderly patients presenting with atypical systemic features. Although elderly individuals often present with non-specific manifestations, the decision to perform L. pneumophila urinary antigen testing in this case was guided by the 2019 IDSA/ATS CAP criteria for severe pneumonia. The patient met three minor criteria-multilobar infiltrates, confusion, and elevated serum urea-fulfilling the definition of severe CAP, for which Legionella testing is specifically recommended [2]. This targeted approach ensures appropriate diagnostic evaluation while avoiding indiscriminate testing in all elderly patients. It emphasizes the importance of maintaining a high index of suspicion, utilizing rapid diagnostics, initiating guideline-directed antimicrobial therapy, and addressing environmental sources to prevent disease transmission. Furthermore, understanding the mechanistic basis of hyponatremia, neurological, and gastrointestinal manifestations can aid clinicians in early recognition and appropriate management of this potentially severe infection.

## Conclusions

This case demonstrates that Legionnaires’ disease can present with predominantly gastrointestinal and neurological manifestations in elderly patients with multimorbidity, even in the absence of respiratory symptoms. Such atypical presentations highlight the diagnostic challenge and the importance of maintaining a high index of suspicion when chest imaging shows infiltrates. Rapid diagnostic modalities, including urinary antigen testing and PCR, are invaluable for confirming infection and guiding early, targeted therapy. Identification of environmental reservoirs, such as hot tubs, is essential for public health control. Environmental investigation and surveillance remain critical to preventing further cases and reducing mortality.
